# Domain-General Enhancements of Metacognitive Ability Through Adaptive Training

**DOI:** 10.1037/xge0000505

**Published:** 2019-01

**Authors:** Jason Carpenter, Maxine T. Sherman, Rogier A. Kievit, Anil K. Seth, Hakwan Lau, Stephen M. Fleming

**Affiliations:** 1 Wellcome Centre for Human Neuroimaging, University College London, and Department of Psychology, University of California, Los Angeles; 2 Sackler Centre for Consciousness Science and School of Engineering and Informatics, University of Sussex; 3 Max Planck University College London Centre for Computational Psychiatry and Ageing Research, University College London, and MRC Cognition and Brain Sciences Unit, University of Cambridge; 4 Sackler Centre for Consciousness Science and School of Engineering and Informatics, University of Sussex; 5 Department of Psychology and Brain Research Institute, University of California, Los Angeles, and Department of Psychology, University of Hong Kong; 6 Wellcome Centre for Human Neuroimaging, and Max Planck University College London Centre for Computational Psychiatry and Ageing Research, University College London

**Keywords:** cognitive training, metacognition, introspection, confidence, longitudinal modeling

## Abstract

The metacognitive ability to introspect about self-performance varies substantially across individuals. Given that effective monitoring of performance is deemed important for effective behavioral control, intervening to improve metacognition may have widespread benefits, for example in educational and clinical settings. However, it is unknown whether and how metacognition can be systematically improved through training independently of task performance, or whether metacognitive improvements generalize across different task domains. Across 8 sessions, here we provided feedback to two groups of participants in a perceptual discrimination task: an experimental group (*n* = 29) received feedback on their metacognitive judgments, while an active control group (*n* = 32) received feedback on their decision performance only. Relative to the control group, adaptive training led to increases in metacognitive calibration (as assessed by Brier scores), which generalized both to untrained stimuli and an untrained task (recognition memory). Leveraging signal detection modeling we found that metacognitive improvements were driven both by changes in metacognitive efficiency (meta-*d*′/*d*′) and confidence level, and that later increases in metacognitive efficiency were positively mediated by earlier shifts in confidence. Our results reveal a striking malleability of introspection and indicate the potential for a domain-general enhancement of metacognitive abilities.

*Metacognition* refers to the ability to monitor and introspect upon cognitive performance. An individual with good metacognition is aware of fluctuations in task performance, and appropriately modulates their confidence level (e.g., holding higher confidence when correct, and lower confidence when incorrect). Although metacognitive abilities are often treated as stable characteristics of individuals (Allen et al., 2017; Fleming, Weil, Nagy, Dolan, & Rees, 2010; McCurdy et al., 2013; Song et al., 2011), several lines of research hint at their malleability. For instance, practicing meditation boosts the accuracy of retrospective confidence judgments about recognition memory decisions (Baird, Mrazek, Phillips, & Schooler, 2014) and monitoring of decision errors can be modulated by drugs (Hester et al., 2012; Hauser et al., 2017) and brain stimulation (Harty et al., 2014). Moreover, recent work has identified distinct neural substrates in the frontal and parietal lobes supporting metacognitive monitoring across a range of tasks (Allen et al., 2017; Baird, Smallwood, Gorgolewski, & Margulies, 2013; Cortese, Amano, Koizumi, Kawato, & Lau, 2016; Fleming et al., 2010; McCurdy et al., 2013; see Fleming & Dolan, 2012, for a review), suggesting the potential for targeted modulation of metacognition independently of changes in first-order performance.

Previous attempts to improve metacognitive ability (confidence calibration) through explicit instruction, practice, feedback, or a combination of these manipulations have led to mixed results, with some studies documenting increases, and others documenting null findings (e.g., Adams & Adams, 1958; Bol, Hacker, O’Shea, & Allen, 2005; Lichtenstein & Fischhoff, 1980; Nietfeld & Schraw, 2002; Renner & Renner, 2001; Sharp, Cutler, & Penrod, 1988). One potential explanation for such heterogeneity of results is that training may impact first-order performance, thus masking subtle changes in metacognition because they are positively correlated (Fleming & Lau, 2014; Sharp et al., 1988). Recent developments in the analysis of confidence-rating data now permit the effective isolation of metacognitive ability (the relationship between performance and confidence) from changes in performance through calculation of the signal detection theoretic parameter meta-*d*′ (Fleming & Lau, 2014; Maniscalco & Lau, 2012). Because meta-*d*′ is in the same units as first-order performance (*d*′) a metacognitive “efficiency” score (meta-*d*′/*d*′) is straightforward to calculate and indexes an individual’s metacognitive capacity with respect to a particular level of task performance. Although training paradigms have proven effective in other cognitive domains, such as working memory (Constantinidis & Klingberg, 2016; Klingberg, 2010; Morrison & Chein, 2011; von Bastian & Oberauer, 2014) and even perceptual domains such as synesthesia (Bor, Rothen, Schwartzman, Clayton, & Seth, 2014), it remains unknown whether metacognitive efficiency can be improved with practice, and whether putative metacognitive training supports transfer to untrained tasks or domains. Given that effective monitoring of performance is deemed important for effective behavioral control (Metcalfe & Finn, 2008; Nelson & Narens, 1990), intervening to improve metacognition may have widespread benefits, for example in educational and clinical settings.

However, it remains unclear whether such an intervention is a priori plausible for alleviating metacognitive deficits, or enhancing baseline metacognitive performance, across a range of scenarios. There is disagreement about the extent to which metacognitive ability is a domain-general resource that can be applied to multiple different tasks, or whether it comprises domain-specific components. Recent findings suggest that confidence is encoded in a “common currency” that can be compared across a range of arbitrary decision scenarios (de Gardelle & Mamassian, 2014; Faivre, Filevich, Solovey, Kuhn, & Blanke, 2017). However other studies indicate a substantial fraction of individual variation in metacognitive ability is domain-specific (Kelemen, Frost, & Weaver, 2000; Morales, Lau, & Fleming, 2018), consistent with dissociable neural correlates of perceptual and memory metacognition (Baird et al., 2013; Fleming et al., 2014; McCurdy et al., 2013; Morales et al., 2018). To the extent to which metacognition is domain-specific, training in one domain (e.g., on the computerized perceptual discrimination task that we use here) may provide only narrow benefits to metacognition in that domain and be of limited value outside the laboratory. To evaluate the potential benefits of training on metacognitive ability it is therefore critical to assess whether such improvements generalize to an untrained task or cognitive domain. A useful parallel can be drawn with the literature on working memory training—here, meta-analysis suggests that “near” transfer to closely related tasks is commonly obtained, but evidence for far transfer is less consistent (Melby-Lervag & Hulme, 2013). The transfer profile of metacognitive training remains unknown.

Here we sought to investigate these questions by providing differential feedback to two groups of participants over eight training sessions on a perceptual discrimination task. A control group received feedback on their objective perceptual discrimination performance, whereas an experimental group received feedback on the calibration of their metacognitive judgments with respect to objective performance. Despite both groups exhibiting similar task performance, the experimental group displayed selective enhancements of metacognitive calibration (the association between confidence and performance) on the trained task. Furthermore, we obtained evidence for a transfer of metacognitive enhancements to an untrained stimulus type and to an untrained task (recognition memory). Together our results reveal a hitherto unreported malleability of domain-general mechanisms supporting metacognition and highlight the potential for generalized improvements in metacognitive ability.

## Method

In this section, we report how we determined our sample size, all data exclusions, all manipulations, and all measures in the study (Simmons, Nelson, & Simonsohn, 2012).

### Participants

We set out to recruit at least 30 participants per group (60 in total), and no data were analyzed prior to completion of data collection. Data were collected via Amazon Mechanical Turk (https://www.mturk.com), an online crowdsourcing platform. *N* = 102 adult participants completed at least the first session of the study. Of these, eight participants were excluded from further training because of floor or ceiling performance in the pretraining baseline session, and a further 25 participants exited the study before completing the full training protocol. Of the remaining 69 participants, one was excluded because of technical problems and seven were excluded on the basis of data quality criteria explained in detail in the following text. Final analyses were carried out on a dataset of 61 participants (35 women, 26 men; *M* age = 38.1 years, age range = 20–64 years). Participants were required to use either Google Chrome or Mozilla Firefox in full-screen mode to complete the experiment on a computer(s) of their choosing.

Before participating in each session, all participants provided informed consent as approved by the University of California, Los Angeles Institutional Review Board (IRB#15–001476). Participants received monetary compensation in U.S. dollars (range = $37.60–$44.60) for approximately 5 hr (*M* = 5.33 hr) of participation over a period of 9 to 35 days (control group: *M* = 15.5 days; experimental group: *M* = 15.4 days; independent samples *t*[59] = 0.10, *p* = .92).

### Procedure

**Figure 1 fig1:**
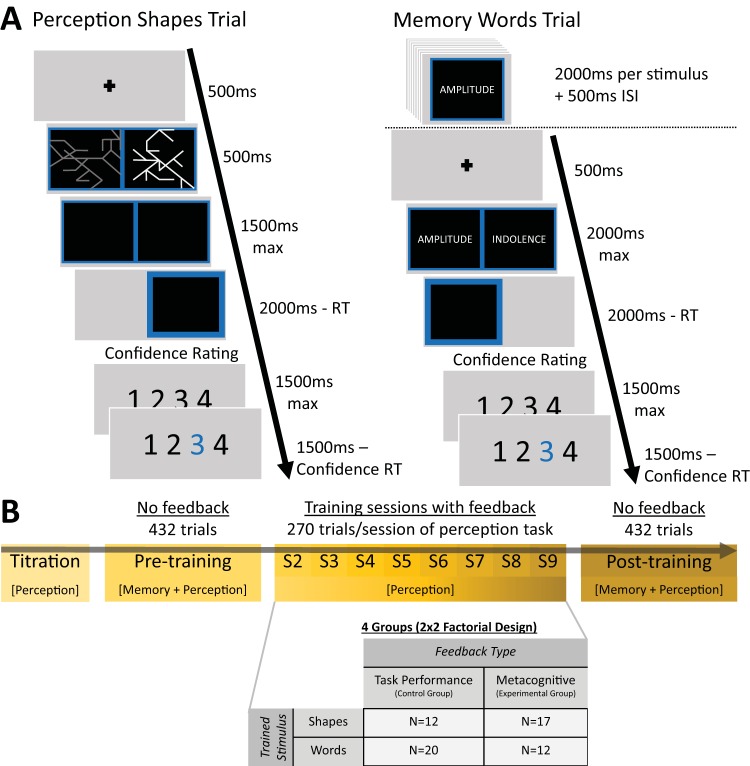
Task and session structure. Panel A: Participants were tested on both a perceptual discrimination and recognition memory task, each involving two stimulus types: abstract shapes and words. The perceptual task (left) comprised a two-alternative forced-choice discrimination judgment as to the brighter of two simultaneously presented stimuli on each trial. The memory task (right) comprised an encoding phase followed by a series of two-alternative forced-choice recognition memory judgments. Panel B: Experiment timeline. Each participant completed 10 sessions in total: a pretraining session, eight training sessions, and a posttraining session. All four conditions were assessed at pre- and posttraining, but only the perceptual task with a single stimulus type (shapes or words) was trained during Sessions 2 through 9. During training sessions, the control groups received feedback on their objective perceptual discrimination performance, whereas the experimental groups received feedback on their metacognitive calibration. In both groups, feedback was delivered every 27 trials.

The experiment was divided into three phases: Phase 1, pretraining (one session) → Phase 2, training (eight sessions) → Phase 3, posttraining (one session), resulting in 10 sessions in total. Figure 1B provides an overview of the experiment timeline. Phase 1 consisted of stimulus titration and a pretraining session to evaluate baseline metacognitive accuracy in a series of two-alternative forced-choice (2AFC) discrimination tasks (see the Task section and Figure 1A). One set of tasks assessed perceptual discrimination, the other set assessed recognition memory. The tasks followed a 2 × 2 factorial design crossing cognitive domain (perception or memory) with stimulus type (explained in detail subsequently). Each task consisted of 108 trials, giving 432 total trials in the pretraining session. The order of these tasks was counterbalanced such that each participant performed both tasks in one domain followed by both tasks in the other domain, and within each domain the order of stimulus types was also counterbalanced.[Fig-anchor fig1]

At the start of Phase 2 participants were assigned to one of four groups. Each group formed a cell in a 2 × 2 factorial design crossing feedback type (control group vs. experimental group) and trained stimulus type (see the Training section to follow). All participants received training on the perceptual task only, with the recognition memory task introduced again at posttraining to assess transfer to a different task domain. During the training phase, each of the eight sessions consisted of 270 trials (2,160 trials total), and block-wise feedback was administered every 27 trials (see the Feedback section to follow).

Phase 3, the final posttraining session, was identical to the pretraining session Phase 1 except that stimulus titration was omitted. Task order was counterbalanced against that used in pretraining, such that each participant performed the task domains (memory, perception) in the opposite order to that seen in pretraining. The order of stimulus types within each domain remained the same.

Phase 1 lasted approximately 60 min, the eight training sessions in Phase 2 lasted approximately 25 min each, and Phase 3 lasted approximately 45 min. Participants were required to wait a minimum of 24 hr between each session and were asked via e-mail to complete each subsequent session within 48 hr to 72 hr of the previous session.

### Tasks

Figure 1A displays example trial timelines for the perception and memory tasks. In the perception task, participants were presented with two images (i.e., words or shapes) and asked to respond to the following question: “Which [image] has brighter lines?” In the memory task, participants were first presented with a series of images to memorize (again, words or shapes). On each subsequent trial, one old image and one novel image were presented with the instruction to respond to the following question: “Which [image] have you seen before?” In all tasks, after each decision, participants were asked to rate their confidence on a four-point scale, whereon 1 = *very low confidence*, 2 = *low confidence*, 3 = *high confidence*, and 4 = *very high confidence*.

In the pretraining session, before beginning each task, participants completed three practice trials to become acquainted with making perception/memory judgments and using the confidence rating scale. Following the practice trials, we probed knowledge of how to perform the perception/memory judgments by asking the following comprehension question: “In the perception/memory task, how do you decide which image to choose?” The three response options were “which one you remember,” “which has more lines,” and “which is brighter.” If a participant answered either question incorrectly, they were excluded from further participation and offered a partial reimbursement determined by the proportion of the session completed. There were no practice trials or comprehension questions in the posttraining session.

### Training

The second phase of the study involved eight training sessions of 270 trials each (2,160 trials in total), spread over 8 to 34 days. Participants were randomly allocated to one of four groups in a 2 × 2 factorial design crossing feedback type (control group vs. experimental group) and trained stimulus type (shapes or words). All groups received block-wise feedback in the form of reward (points) every 27 trials. The control groups (for both stimulus types) received feedback on their objective perceptual discrimination performance; the experimental groups (for both stimulus types) received feedback on their metacognitive calibration, as determined by the average quadratic scoring rule (QSR) score. The QSR provides a metric for how closely confidence ratings track accuracy (Staël von Holstein, 1970) and is equal to one minus the Brier score (Fleming & Lau, 2014). The rule underpinning each feedback type is described in more detail in the following Feedback section.

To ensure that each group fully understood how points could be earned, instructions were provided on the meaning of the feedback schedule. Participants completed eight demonstration trials which explained how earnings changed based on their objective performance (control group) or the correspondence between confidence and accuracy (experimental group). After the demonstration, participants performed 10 practice trials in which they received full feedback and a brief explanation. Note that in the demonstration and practice trials, feedback was calculated on a trial-by-trial basis and therefore differed from the block-wise feedback received in the training sessions (see the following Feedback section). After the demonstration and practice trials, participants were asked two comprehension questions probing their understanding of how to earn points. If they failed these questions they were asked to attempt them again until they were successful.

### Task Performance Titration

Throughout the entire 10-session experiment, the performance of each participant was titrated online to achieve approximately 75% correct for all tasks except the memory-words task. This “threshold” level of percent correct produces sufficient trials for each signal detection theory outcome (hits, misses, false alarms and correct rejections) for analysis of *d*′ and meta-*d*′ (Maniscalco & Lau, 2012), and ensured any changes in metacognitive sensitivity were not confounded by shifts in task performance.

Titration was accomplished in different ways for each task. In the perception tasks (for both word and shapes), we implemented two interleaved, weighted and transformed staircase procedures on the brightness of the images. We alternated two staircases with differently weighted step sizes. In the first staircase, after two consecutive correct responses the stimulus brightness was decreased by two steps; after one incorrect response the brightness was increased by four steps. In the second staircase, after three correct responses the brightness level was decreased by three steps, after 1 incorrect response the brightness was increased by four steps. Note that these are not traditional *n*-down/one-up procedures as the correct trial counter was not reset to zero after each pair or triplet of correct responses. However, we found in pilot work that this interleaved method stably converges to 75% correct. Brightness levels were adjusted independently for word and shape stimuli. In order to define initial brightness levels, participants performed a 60-trial titration block for each stimulus type after the practice trials and before beginning the pretraining session. The final brightness level at the end of the titration block acted as the initial brightness level for pretraining Session 1. Each subsequent Session 2 to 10 began with the final brightness level of the previous session.

In the memory-shapes task, the number of stimuli in the encoding period was adjusted based on the average percent correct recorded over the previous two blocks. If average performance exceeded 75% correct, one additional image was added to the encoding set. If performance dropped below 70% correct, one image was removed, down to a minimum of two images. We initialized the encoding set size at four images. Note that even though the minimum set size was two, the underlying staircase value had no minimum value.

For the memory-words task, we employed a fixed set size of 54 words. This larger set size was based on initial pilot data and the procedure of McCurdy et al. (2013) and reflects the fact that participants typically find encoding and remembering individual words significantly easier than encoding and remembering abstract shapes.

### Feedback

Feedback in the form of points was given based on task performance in the control group and metacognitive calibration in the experimental group. We rewarded the control group on their achieved difficulty level, specified as the inverse distance between the current brightness level and the minimum brightness of 128:

difficulty=128−(brightness−128)

where brightness level ϵ [128 − 256] → difficulty level ϵ [0 − 128]. We chose difficulty level instead of accuracy as the relevant performance measure because accuracy was titrated to ∼75% correct in each block.

We rewarded the experimental group using the QSR. The QSR is a proper scoring rule in the formal sense that maximum points are obtained by jointly maximizing the accuracy of choices and confidence ratings (Staël von Holstein, 1970). We mapped each confidence rating onto a subjective probability correct using a linear transformation: *p*(correct)= −1/3 + confidence/3, where the confidence rating is ϵ [1 − 4] → *p*(correct) ϵ [0 − 1]. On each trial, *i* the QSR score is then obtained as follows:

$$QSR_{i}=1-{\left(accuracy_{i}-p{\left(correct\right)}_{i}\right)}^{2}$$

where accuracy is ϵ [0, 1] and *p*(correct) ϵ [0 − 1] → QSR ϵ [0 − 1]. This rule ensures that people receive the highest number of points when they are highly confident and right, or unconfident and wrong (i.e., metacognitively accurate).

Despite feedback in each group being based on different variables, we endeavored to equate the distribution of points across groups. We used data from an initial pilot study (without feedback) to obtain distributions of expected difficulty level and QSR scores. We then calculated the average difficulty level/QSR score for each block, and fit Gaussian cumulative density functions (CDFs) to these distributions of scores. These CDFs were then used to transform a given difficulty or QSR score in the main experiment to a given number of points.

### Compensation

Participants were compensated at approximately $4 per hr, plus a possible bonus on each session. Base pay for the 60-min pretraining session was $4, for the eight 25-min training sessions $2 each, and for the 45-min posttraining session $3. Participants were informed they had the opportunity to earn a session bonus if they outperformed a randomly chosen other participant on that session. In practice, bonuses were distributed pseudorandomly to ensure equivalent financial motivation irrespective of performance. All participants received in the range of four to seven bonuses throughout the course of the 10-session study. Bonuses comprised an additional 70% of the base payment received on any given session.

In addition to the pseudorandom bonuses, all participants received a $3 bonus for completing half (5) of the sessions and a $6 bonus for completing all (10) of the sessions. Total earnings ranged from $37.60 to $43.90 across participants, and income did not differ significantly between groups (control group: *M* = $41.47; experimental group: *M* = $40.98; *t*[59] = 0.94, *p* = .35). The base payment was paid immediately after completing each session and accumulated bonuses were paid only if the participant completed the full 10 session experiment.

### Quantifying Metacognition

Our summary measure of metacognitive calibration was the QSR score achieved by participants before and after training. To separately assess effects of training on metacognitive bias (i.e., confidence level) and efficiency (i.e., the degree to which confidence discriminates between correct and incorrect trials), we also fitted meta-*d*′ to the confidence rating data. The meta-*d*′ model provides a bias-free method for evaluating metacognitive efficiency in a signal detection theory framework. Specifically, the ratio meta-*d*′/*d*′ quantifies the degree to which confidence ratings discriminate between correct and incorrect trials while controlling for first-order performance (*d*′). Using this ratio as a measure of metacognition effectively eliminates performance and response bias confounds typically affecting other measures (Barrett, Dienes, & Seth, 2013; Fleming & Lau, 2014). We conducted statistical analyses on log(meta-*d*′/*d*′) as a logarithmic scale is appropriate for a ratio measure, giving equal weight to increases and decreases relative to the optimal value of meta-*d*′/*d*′ = 1.

Meta-*d*′ was fit to each participant’s confidence rating data on a per-session basis using maximum likelihood estimation as implemented in freely available MATLAB code (http://www.columbia.edu/~bsm2105/Type2sdt/). Metacognitive bias was assessed as the average confidence level across a particular task and session, irrespective of correctness.

### Analysis Plan

By employing a combination of frequentist and Bayesian statistics, we aimed to assess the differential impact of the training manipulation across groups and the transfer of training effects across domains. To model the dynamics of training, we additionally assessed the drivers of the training effect using latent change score modeling and mediation analysis.

We first applied mixed-effects analyses of variance (ANOVAs) to measures of metacognition including group as a between-subjects factor and task domain as a within-subjects factor. Complementary to classical ANOVAs, we also used a Bayesian “analysis of effects” that quantifies evidence in support of transfer of training effects across stimulus types and domains. Evidence in support of transfer is indicated by a simpler model, without stimulus or domain interaction terms, providing a better fit to the data. Finally, by modeling our data using latent changes scores, we gained insight into whether effects of training are dependent on baseline metacognitive abilities. In addition, we used mediation modeling to ask whether early shifts in confidence strategy facilitated later improvements in introspective ability.

#### Analysis of effects of training

In addition to the pretraining exclusion criteria detailed in the preceding text, the following set of predefined exclusion criteria was applied after data collection was complete. One participant was excluded for performing outside the range of 55% to 95% correct in at least one condition/session. One participant was excluded due to their average difficulty level calculated across all sessions dropping below 2.5 standard deviations below the group mean difficulty level. Five participants were excluded for reporting the same confidence level on 95% of trials for three or more sessions. Finally, trials in which either the participant did not respond in time (response times >2,000 ms) or response times were less than 200 ms were omitted from further analysis (0.98% of all trials).

To evaluate effects of training, we compared data from the pre- and posttraining sessions using mixed-model ANOVAs in JASP (https://jasp-stats.org/) to assess the presence of training effects as a function of domain and stimulus type (factors: Training × Domain × Stimulus × Group). We coded the stimulus factor in terms of whether the stimulus encountered during the pre- and posttraining sessions was trained or untrained. We also used a Bayesian “analysis of effects” in JASP to quantify evidence for and against across-stimulus and across-domain transfer of training effects on confidence and metacognitive efficiency (Rouder, Morey, Speckman, & Province, 2012).

#### Latent change modeling

To assess the dependence of training gains in the (trained) perceptual domain and the (untrained) memory domain on baseline metacognitive abilities, we fit a bivariate latent change score (BLCS) model to QSR scores (Kievit et al., 2017; McArdle & Nesselroade, 1994). LCS models conceptualize differences between pre- and posttraining performance as latent change factors. The basic equation of the LCS model specifies the score of individual *i* in domain *Y* at posttraining as a sum of the score at pretraining and a change, or difference, score:

$$Y_{i.post}=\beta_{i.pre}Y_{i.pre}+\Delta Y_{i}.$$

By setting the regression weight β_*i.pre*_ to 1, change scores can be rewritten as follows:

$$\Delta Y_{i}=Y_{i.post}-Y_{i.pre}.$$

This formulation allows the change score for memory or perceptual metacognitive calibration (e.g., Δ*M* or Δ*P*) itself to be modeled as being dependent on two influences, a self-feedback process β and a coupling process γ:

$$\Delta M_{i}=\beta_{M}M_{i.pre}+\gamma_{M}P_{i.pre},$$

$$\Delta P_{i}=\beta_{P}P_{i.pre}+\gamma_{P}M_{i.pre},$$

where *P* and *M* denote the QSR scores for the perceptual and memory domains, respectively. To simplify the model, we included only data from the trained stimulus type in both domains. The self-feedback parameters (β) are assumed to reflect a combination of regression to the mean, potential dependence of training on baseline performance (e.g., the extent to which training gains are greater for individuals with low/high baseline calibration) and/or ceiling effects. The coupling parameters (γ) assess the extent to which change in one domain is dependent upon baseline calibration in the other domain, above and beyond the effects of self-feedback. The bivariate LCS formulation also allows estimation of the extent of correlated change, reflecting the degree to which training effects co-occur across domains, having taken into account the coupling and self-feedback parameters.

Models were estimated in the lavaan package for R (Version 5.23; Rosseel, 2012) using full information maximum likelihood, robust (Huber-White) standard errors and a scaled test statistic. We assessed overall model fit via the root-mean-square error of approximation (RMSEA; acceptable fit: < 0.08; good fit: < 0.05), the comparative fit index (CFI; acceptable fit: 0.95 to 0.97; good fit: > 0.97) and the standardized root-mean-square residual (SRMR; acceptable fit: 0.05 to 0.10, good fit: < 0.05; Schermelleh-Engel, Moosbrugger, & Muller, 2003).

#### Analysis of training dynamics

To investigate the dynamics of the training effect we calculated objective performance, metacognitive bias and metacognitive efficiency separately for each of the eight training sessions. This allowed us to visualize any progressive effects of feedback on metacognition while also establishing the stability of task performance during training sessions. To assess whether shifts in metacognitive bias mediate the impact of training on metacognitive efficiency, we fit mediation models using the Mediation Toolbox for MATLAB (https://github.com/canlab/MediationToolbox). The Mediation Toolbox uses nonparametric bootstrapping, which is more robust in handling violations to normality than traditional parametric approaches such as the Sobel test.

## Results

To quantify effects of training on both performance and metacognition, we conducted mixed-model ANOVAs comparing pre- and posttraining sessions (factors: Training × Domain × Stimulus × Group). We coded the stimulus factor in terms of whether the stimulus encountered during pre- and posttraining was trained or untrained.

### First-Order Performance

**Figure 2 fig2:**
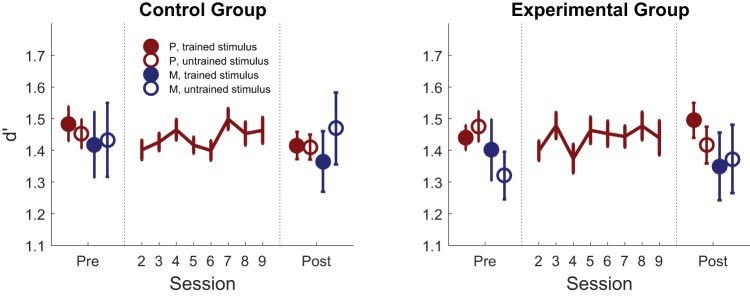
First-order discrimination performance. Effect of training on first-order performance (*d*′) in the control group (who received feedback on perceptual discrimination performance) and the experimental group (who received feedback on their metacognitive judgments) as a function of whether the judgment was made on a perception (red) or memory (blue) trial, and on the trained (filled) or untrained (unfilled) stimulus type. Error bars represent between-subjects *SEM*. P = perception; M = memory.

Task performance (*d*′) was stable across pre- and posttraining sessions in both groups (main effect of training: *F*[1, 59] = 0.34, *p* = .56), and both groups performed similarly (main effect of group: *F*[1, 59] = 0.15, *p* = .71), as expected from the staircase procedure (see Figure 2). When examining task difficulty (brightness level, controlled by the staircase procedure), we found that both groups achieved a higher difficulty level (lower brightness level) following training (main effect of training: *F*[1, 59] = 15.2, *p* < .001), with a trend toward a more prominent difference in the control group who received feedback on this quantity (training_control_: *F*[1, 31] = 16.46, *p* < .001; training_experimental_: *F*[1, 28] = 2.23, *p* = .15; Training × Group: *F*[1, 59] = 3.14, *p* = .081; see online supplemental Figure S1).[Fig-anchor fig2]

### Metacognitive Calibration

**Figure 3 fig3:**
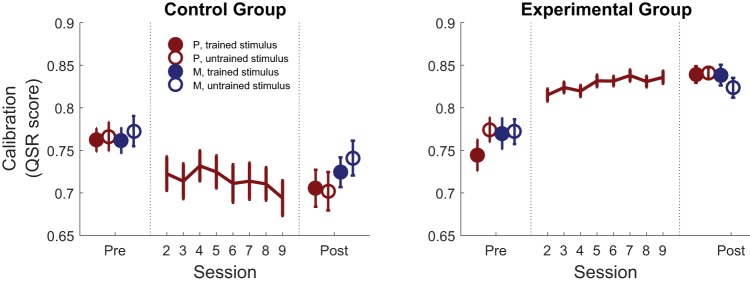
Metacognitive calibration. Effect of training on confidence calibration (the average quadratic scoring rule score [QSR]). Calibration improved over training sessions in the experimental group in the absence of changes in first-order performance, and this improvement transferred both to an untrained stimulus and untrained recognition memory task. Error bars represent between-subjects *SEM.* P = perception, M = memory.

To quantify metacognitive calibration before and after training we examined the average score achieved from the QSR. QSR scores are highest when confidence matches accuracy on a trial-by-trial basis—that is, when participants report higher confidence after correct trials, and lower confidence after errors. Critically, we observed a significant Training × Group interaction, *F*(1, 59) = 38.07, *p* < .001, driven by a robust increase in calibration in the experimental group, *F*(1, 28) = 25.55, *p* < .001, and a decrease in the control group, *F*(1, 31) = 13.15, *p* = .001 (see Figure 3 and online supplemental Figure S2).[Fig-anchor fig3]

Having revealed a selective improvement in metacognitive calibration in the Experimental group, we next asked whether this improvement generalized across stimulus types or domains. To quantify the evidence for and against across-stimulus and across-domain transfer, we performed Bayesian ANOVAs (see Table 1) on QSR scores in the experimental group. This approach (known as an *analysis of effects*; Rouder et al., 2012) analyzes all possible models of the data (e.g., main effects only, main effects + interaction effect). For each effect, a Bayes factor quantifies the degree to which the data support models including versus excluding that effect. We found evidence in support of modeling a main effect of training (*BF_inclusion_* = 1.1 × 10^10^) and evidence against modeling Training × Stimulus (*BF_inclusion_* = 0.13) and Training × Domain (*BF_inclusion_* = 0.10) interactions (see Table 1). In other words, the best-fitting model is one in which the training effect on QSR scores was similar for both stimulus types (shapes and words) and both task domains (perception and memory), supporting both transfer to the untrained stimulus (within the trained perceptual task) and transfer to the recognition memory task, for both stimulus types. Together these results show that our metacognitive feedback protocol was able to selectively improve the correspondence between confidence and accuracy when feedback was removed, and that this improvement in confidence estimation transferred both to an untrained stimulus type and an untrained task (recognition memory).[Table-anchor tbl1]

### Metacognitive Efficiency and Bias

Recent approaches distinguish between two key aspects of metacognitive performance (Fleming & Lau, 2014). The first is efficiency: How accurately do participants discriminate between correct and incorrect trials for a given level of first-order task performance? The second is bias: Are participants generally more or less confident in a particular task or condition? Using a signal detection theory approach, we sought to reveal whether metacognitive improvements due to training were due to changes in efficiency, bias or both. The ratio meta-*d*′/*d*′ quantifies the efficiency with which confidence ratings discriminate between correct and incorrect trials while controlling for first-order performance (*d*′; Maniscalco & Lau, 2012). Bias was assessed as the average confidence level irrespective of whether a trial was correct or incorrect.

**Figure 4 fig4:**
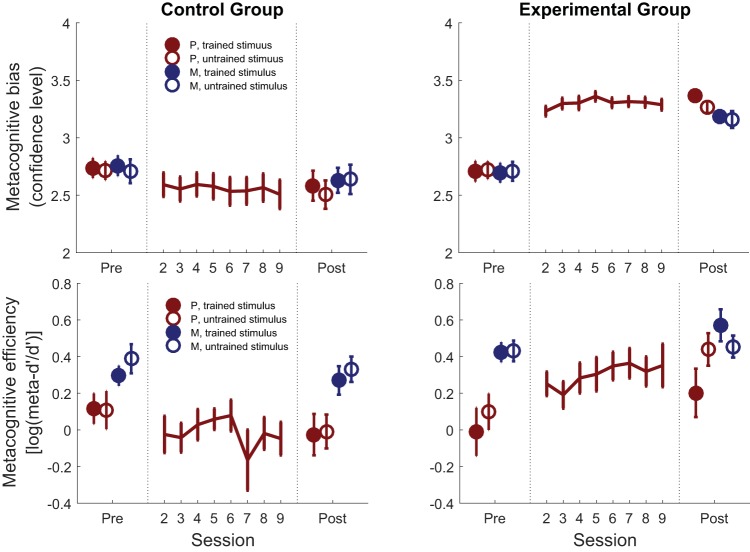
Effects of training on components of metacognition. Effects of training on metacognitive bias (confidence level; top panels) and metacognitive efficiency (log[meta-*d*′/*d*′]; bottom panels). The left-hand column shows data from the control group; the right-hand column shows data from the experimental group. Metacognitive efficiency (log[meta-*d*′/*d*′]) gradually improved over training in the experimental group (bottom panel) in the absence of changes in first-order performance. Error bars represent between-subjects *SEM*. One participant was excluded when plotting mean log(meta-*d*′/*d*′) for Session 6 due to a negative value of meta-*d*′ precluding a log-transform. P = perception, M = memory.

When analyzing metacognitive efficiency (log[meta-*d*′/*d*′]), we observed a significant Training × Group interaction, *F*(1, 59) = 6.96, *p* = .011, driven by a selective increase from pre- to posttraining in the experimental group (training_experimental_: *F*[1, 28] = 6.72, *p* = .015; training_control_: *F*[1, 31] = 1.39, *p* = .25; bottom row of Figure 4). Improvements in metacognitive efficiency were also accompanied by an overall increase in metacognitive bias (confidence level; training_experimental_: *F*[1, 28] = 73.87, *p* < .001; training_control_: *F*[1, 31] = 3.77, *p* = .061; Training × Group: *F*[1, 59] = 49.35, *p* < .001; see the top row of Figure 4).[Fig-anchor fig4]

In a Bayesian analysis of effects, we found positive evidence against the inclusion of a Training × Stimulus interaction term for both metacognitive bias and metacognitive efficiency (see Table 1). In other words, the best-fitting model was one in which the training effect was similar for both stimulus types, supporting the existence of transfer to the untrained stimulus. However, there was equivocal evidence for or against transfer across domains (the Training × Domain interaction term) for both metacognitive bias and metacognitive efficiency, suggesting our data cannot support or refute domain-general training effects when examining these components separately.

### Latent Change Modeling

To identify potential drivers of improvements in metacognitive calibration, we fit bivariate latent change score (LCS) models to the QSR score data. Specifically, we examined the interrelationship between changes in calibration for perception and memory from pretraining (T1) to posttraining (T2; restricted to scores obtained for the trained stimulus type). We assessed the evidence for five possible parameters in the model. First, does baseline perceptual calibration predict the degree of change in perceptual calibration (self-feedback parameter) and/or memory calibration (coupling parameter)? Similarly, does baseline memory calibration predict the degree of change in memory calibration (self-feedback parameter) and/or perceptual calibration (coupling parameter)? Finally, is there evidence for correlated improvements (covariance of change) in perceptual and memory calibration across individuals?

Before fitting the bivariate model, we first fitted two univariate LCS models to each domain separately. In these models, the mean and variance of pretraining scores was constrained to be equal between the experimental and control groups. The memory model fitted the data well: χ^2^(2) = 0.72, *p* = .70; RMSEA < 0.001, 90% confidence interval (CI) [0.000, 0.265]; CFI = 1.000; SRMR = 0.083. The equivalent perceptual model revealed a poor model fit, χ^2^(2) = 2.43, *p* = .30; RMSEA = 0.084, 90% CI [0.000, 0.380]; CFI = 0.91; SRMR = 0.132, which further examination indicated was driven by a higher variance of pretraining QSR scores in the experimental compared with the control group. Allowing the variance of T1 scores to differ between groups restored good model fit: χ^2^(1) = 0.62, *p* = .43; RMSEA < 0.001, 90% CI [0.000, 0.439]; CFI = 1.000; SRMR = 0.046. We thus allowed perceptual T1 variance to differ between groups in the bivariate LCS model considered below. As expected, both univariate models showed evidence for positive change in QSR scores for the Experimental group (unstandardized change score intercepts—perception: 0.80, *SE* = 0.067, *z* = 11.9; memory: 0.64, *SE* = 0.086, *z* = 7.50) but not the control group (perception: 0.19, *SE* = 0.17, *z* = 1.10; memory: 0.089, *SE* = 0.10, *z* = 0.87).^1^

**Figure 5 fig5:**
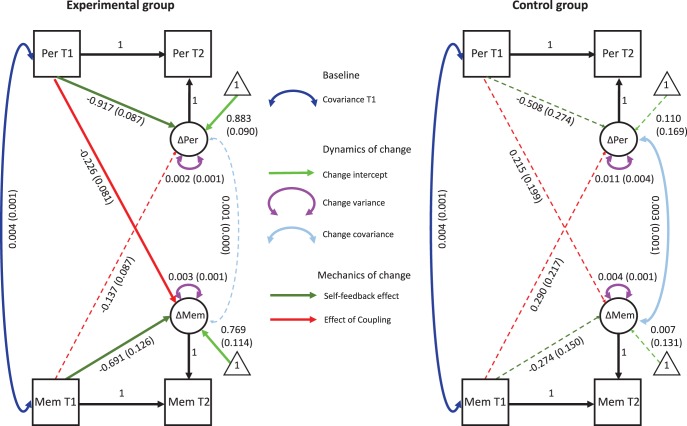
Estimated parameters for the bivariate latent change score model of metacognitive calibration (quadratic scoring rule [QSR] scores). Calibration scores were modeled pretraining (T1) and posttraining (T2) across both domains, restricted to the trained stimulus type. Unstandardized parameter estimates are given separately for each group (with standard errors in parentheses). Solid lines indicate parameter significance at *p* < .05. Note that the T1 covariance, T1 intercepts and T1 memory variance were constrained to be equal across groups. T1 perception variance was estimated separately for each group as explained in the text. Per = perception; Mem = memory.

We next tested for interrelationships between perception and memory calibration in a bivariate LCS model (shown graphically in Figure 5; significant paths are shown as thicker lines). The bivariate LCS model showed good model fit: χ^2^(4) = 3.20, *p* = .53; RMSEA < 0.001, 90% CI [0.000, 0.247]; CFI = 1.000; SRMR = 0.071. Fitted model parameters are shown separately for the control and experimental groups in Figure 5. In addition to the expected significant latent change intercepts in the Experimental group (i.e., increasing scores), the self-feedback parameters were also positive in the Experimental group for both perception and memory, indicating that greater gains in response to training were found in individuals who started off with low metacognitive ability. Notably self-feedback effects were not observed in the Control group, indicating that this pattern of results is unlikely to be due to regression to the mean or repeated testing (constraining coupling and self-feedback parameters to be equal across groups led to a significantly worse model fit; Δχ^2^(4) = 21.16, *p* < .001. The coupling parameter from perception at T1 to memory at T2 was also negative—individuals who started out lower in perceptual calibration improved more on memory calibration, over and above any effect of the self-feedback parameters. Finally, there was no evidence for correlated change between domains in the Experimental group. Together this analysis indicates that effects of metacognitive training depend on baseline metacognitive abilities, both within and across domains.[Fig-anchor fig5]

### Dynamics of Metacognitive Bias and Efficiency

**Figure 6 fig6:**
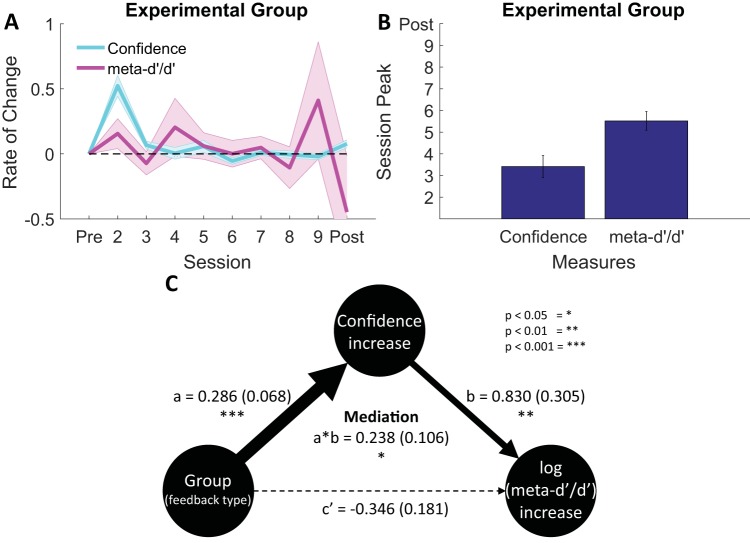
Temporal dissociation of shifts in metacognitive bias and metacognitive efficiency. Panel A: Rate of change over sessions of confidence level and meta-*d*′/*d*′ in the experimental group showing an early shift toward responding with higher confidence. This shift in confidence was dissociated in time from a more gradual improvement in metacognitive efficiency, with the largest changes occurring toward the end of training. Panel B: The session at which this peak shift occurred was significantly earlier for metacognitive bias (confidence level) compared with metacognitive efficiency (meta-*d*′/*d*′). Panel C: Early increases in confidence mediate the impact of feedback type on later increases in metacognitive efficiency. Values outside of parentheses indicate the coefficient mean and values inside parentheses indicate the *SEM*.

Figure 4 indicates that a shift in metacognitive bias (confidence level) in the experimental group occurred immediately on the first training session (see also online supplemental Figure S3), whereas metacognitive efficiency (meta-*d*′/*d*′) increased more gradually over the eight training sessions. To further quantify differences in these time courses we calculated the session-to-session change in confidence and metacognitive efficiency (see Figure 6A). The peak change in confidence was reliably earlier than the peak change in efficiency (see Figure 6B; *t*[28] = 3.67, *p* = .001). To assess whether early changes in confidence were associated with later shifts in metacognitive efficiency, we fit a mediation model (see Figure 6C). Consistent with such a hypothesis, the impact of feedback type (i.e., group) on increases in log(meta-*d*′/*d*′) was positively mediated by initial shifts in confidence, *t*(58) = 2.24, *p* = .028.[Fig-anchor fig6]

## Discussion

Here we reveal a domain-general enhancement of metacognitive abilities despite objective performance (*d*′) remaining unchanged across two distinct perceptual and memory tasks. These changes were only observed when feedback was targeted to metacognitive judgments—an active control group who performed the same tasks but received feedback on first-order (objective) performance did not show the same improvement. Since feedback and financial incentives were matched across groups, motivational factors are unlikely to account for our results. Our findings are instead consistent with a specific effect of metacognitive feedback in enhancing participants’ ability to introspect about self-performance.

In addition to a main effect of training on a trained stimulus type, we obtained evidence that improvements in calibration scores generalized both to other instances of brightness discrimination and, more importantly, an untrained task (recognition memory). This result indicates that the feedback individuals receive on their confidence-accuracy relationship on one task can lead to improved confidence calibration for unrelated tasks, after feedback is removed. Current evidence for a shared neurocognitive resource for metacognition is ambiguous, partly due to a difficulty of distilling metacognitive processes from those supporting primary task performance (Ais, Zylberberg, Barttfeld, & Sigman, 2016; Baird et al., 2013; McCurdy et al., 2013; Song et al., 2011). The observation of domain-general enhancement provides a novel perspective on this issue, suggesting the existence of generic metacognitive resources that can be altered through training. Previous work has suggested confidence estimates are compared in a “common currency” across a range of decision scenarios (de Gardelle & Mamassian, 2014; Faivre et al., 2017), and training may boost the fidelity of such shared signals. In turn our findings hold promise for the future development of training protocols to boost metacognition in applied settings, in which administering domain-specific adaptive training protocols may facilitate improvements in metacognitive abilities more generally.

Latent change score modeling of QSR scores indicated that baseline performance in both trained and untrained tasks (perception and memory) predicted the extent of training gains, with lower baseline levels in a particular domain predicting greater training gains in that domain. In addition, there was evidence for a cross-domain coupling in which lower initial scores on the trained (perceptual) task predicted greater gains in the untrained memory task, over and above effects of self-feedback. These effects were not observed in the active control group, making explanations of such dynamics in terms of regression to the mean or repeated practice less likely. Interestingly a similar pattern has been observed in the literature on working memory training, with the largest training gains observed for those initially low in WM capacity (Zinke, Zeintl, Eschen, Herzog, & Kliegel, 2012, 2014; although see Bissig & Lustig, 2007). Such findings are potentially consistent with initially low performing individuals having a larger (underused) latent potential for WM/metacognition, therefore leading to a stronger response to training. A less interesting explanation is that there are ceiling effects on potential QSR scores, leading to a natural slowdown in gains as a function of starting point. Future work (for instance examining the effects of training over multiple time points, and/or with larger *N* to more precisely estimate the dynamics and cover a wider range of ability levels) is needed to disentangle these possibilities.

We also examined how two key components of metacognition—metacognitive efficiency (meta-*d*′/*d*′) and metacognitive bias (confidence level)—evolved over the course of training. For both components, we observed significant effects of training in the experimental group. However, when examining transfer for each component individually, the picture was more mixed than for the composite calibration measure: while both components generalized to other instances of brightness discrimination, there was equivocal evidence for across-domain transfer to memory metacognition. This pattern of results is potentially consistent with a domain-specificity of metacognitive efficiency for perception versus memory (Baird et al., 2013; Fleming et al., 2014; McCurdy et al., 2013), and recent observations that metacognitive efficiency, while stable within a particular participant across sessions, may be idiosyncratic to particular tasks (Ais et al., 2016). However, we note initial metacognitive efficiency scores for the memory task were high, potentially leading to a ceiling effect on subsequent improvement in this domain. In addition, it remains to be determined whether enhancements of perceptual metacognitive efficiency are limited in transfer to other features within the same modality (such as visual contrast and orientation; Song et al., 2011) or also generalize to other perceptual modalities, such as audition (Faivre et al., 2017).

The time course of training effects provides insight into potential mechanisms supporting metacognitive improvement. Although confidence levels increased during the very first training session and remained stable throughout the remainder of the experiment, metacognitive efficiency climbed more gradually across the eight training sessions. One possible account of this pattern (supported by a mediation analysis) is that an initial shift in confidence strategy facilitates later increases in metacognitive efficiency allowing, for instance, higher confidence to be effectively targeted to correct trials (see online supplemental Figure S2). An implicit signal of whether a first-order decision is likely to be correct may then gradually become associated with higher confidence reports over time, and reinforced by the feedback schedule.

It is important to note that an initial shift in confidence bias does not necessarily reflect a change in metacognition, and may instead reflect a strategic shift in response to the onset of feedback protocol and instructions. Critically, however, such a strategic shift alone is unlikely to explain later change in metacognitive efficiency. To establish the expected impact of a nonspecific bias on measures of metacognitive efficiency, we conducted numerical simulations in which the pretraining confidence data were shifted to create an artificial bias in confidence level (see online supplemental Figure S6). These simulations show that “learning” to increase mean confidence leads to an increase in calibration score, as expected, but is insufficient to produce the observed increases in metacognitive efficiency. Indeed, when confidence bias is artificially induced, metacognitive efficiency is expected to be lower post- compared with pretraining—precisely the opposite of what we find. Thus we believe that these simulations lend support to a conclusion that metacognitive efficiency is specifically increased following feedback on metacognitive judgments, and this effect is not a trivial consequence of strategic biases in confidence.

Our work goes significantly beyond previous attempts to improve the resolution or calibration of confidence judgments. Adams and Adams (1958), Lichtenstein and Fischhoff (1980), and Sharp et al. (1988) reported changes in the confidence-accuracy relationship for participants who received feedback on the correctness of their confidence ratings but lacked active control groups or controls for changes in performance (although Sharp et al., 1988, were aware of this issue). Indeed, participants in the feedback condition of Adams and Adams (1958) reported feeling markedly more enthusiastic about the experiment, suggesting motivation differences may have confounded effects of feedback. Here we addressed this concern by matching feedback schedules and first-order performance levels between the experimental group and an active control group, who received equivalent feedback directed at first-order performance. Intriguingly, the feedback protocol implemented in the present study may represent one among many possible methods for inducing increases in metacognitive efficiency (Lebreton et al., 2018). Other feedback protocols may operate via a different mechanism, for example, learning to decrease error trial confidence, rather than increasing one’s confidence in being correct. Future work could investigate the scope of possible training protocols by manipulating parameters such as titrated performance level and feedback schedule.

Fine-grained introspective ability is useful for several reasons. First, it aids the control of task performance—becoming aware of making suboptimal choices is a useful signal for prompting changes of mind (Folke, Jacobsen, Fleming, & De Martino, 2016) and for the guidance of learning (Metcalfe & Finn, 2008; Nietfeld & Schraw, 2002; Purcell & Kiani, 2016). Second, appropriate sensitivity to self-performance is important when interacting with others (Bahrami et al., 2010; Shea et al., 2014), allowing communication of degrees of belief to improve group decision-making and avoid overconfident testimony (e.g., in an eyewitness context; Busey, Tunnicliff, Loftus, & Loftus, 2000). Finally, metacognition is a potential target of interventions in psychiatric disorders including schizophrenia and depression (Moritz & Woodward, 2007). Developing tools to improve metacognitive abilities may therefore have widespread impact in a variety of settings. Here, despite obtaining evidence for generalization to an untrained task, such “transfer” was limited to a suite of computerized, two-alternative forced choice tasks with confidence ratings. Further work is needed to assess whether metacognitive training has more widespread benefits for unrelated tasks and/or for learning contexts that place demands on metacognitive control.

Our results open up new questions regarding the nature of the malleability of metacognition displayed in the present study. Specifically, the duration and generality of improvements in introspective abilities remain to be determined. We might expect improvements in the ability to introspect about self-performance to be accompanied by changes in brain structure, function, and/or connectivity within frontoparietal networks previously implicated in supporting metacognition (Allen et al., 2017; Baird et al., 2013; Cortese et al., 2016; Fleming & Dolan, 2012; Fleming et al., 2010; McCurdy et al., 2013). A distinction has recently been drawn between lower-level (and potentially generic) signals of confidence and higher-order elaboration of such signals for use in communication and control (Fleming & Dolan, 2012; Morales et al., 2018). By combining the current behavioral intervention with neuroimaging measures it may be possible to determine whether one or both of these levels of processing are affected by metacognitive training. Ongoing work in our laboratory is tackling this question.

## Supplementary Material

10.1037/xge0000505.supp

## Figures and Tables

**Table 1 tbl1:** Bayesian ANOVA Analysis of Effects

Effects	Calibration (QSR)		Metacognitive bias (confidence level)		Metacognitive efficiency log (meta-*d*′/*d*′)	
	BF_inclusion_	Evidence	BF_inclusion_	Evidence	BF_inclusion_	Evidence
Training	1.09e+10	Very strong for	∞	Very strong for	5.55	Positive for
Domain	.08	Positive against	.46	Insubstantial	2348.77	Very strong for
Stimulus	.09	Positive against	.08	Positive against	.20	Positive against
Training × Domain	.10	Positive against	.59	Insubstantial	1.18	Insubstantial
Training × Stimulus	.13	Positive against	.09	Positive against	.13	Positive against
Domain × Stimulus	.01	Strong against	.04	Strong against	.46	Insubstantial
Training × Domain × Stimulus	3.66e-4	Very strong against	4.55e-4	Very strong against	.07	Positive against
*Note*. Evidence in support of including different explanatory variables in models of metacognitive calibration in the experimental group. We obtained positive evidence against inclusion of a Training × Stimulus interaction term for all measures, indicating the best-fitting model is one in which the training effect is similar for both stimulus types. There was positive evidence against inclusion of a Training × Domain interaction term (indicating transfer across domains) in models of calibration (quadratic scoring rule [QSR] score) and equivocal evidence for or against this term in models of both metacognitive bias and metacognitive efficiency. Strength of evidence is evaluated using Kass and Raftery’s (1995) interpretation of the Bayes factor. ANOVA = analysis of variance; BF = Bayes factor.						

## Footnotes

1 Note that these intercept parameters can be interpreted only in the context of the full LCS model that includes the self-feedback pathway.
