# Supplementary material for: Domain-General Enhancements of Metacognitive Ability Through Adaptive Training
Source: J Exp Psychol Gen. 2019 Jan;148(1):51–64. doi: 10.1037/xge0000505 (PMC6390881; doi:10.1037/xge0000505)
Supplement: Supplementary file 1 [file XGE20170607R1.pdf]

SUPPLEMENTAL INFORMATION

Domain-general enhancements of metacognitive ability through adaptive training

Jason Carpenter, Maxine T. Sherman, Rogier A. Kievit, Anil K. Seth, Hakwan Lau, Stephen M. Fleming*

*Correspondence: stephen.fleming@ucl.ac.uk

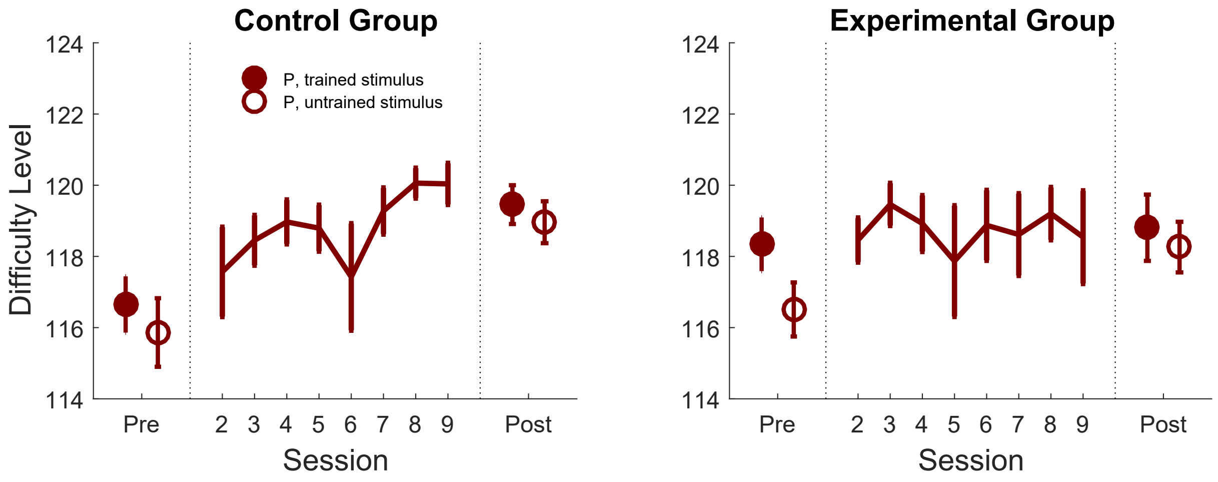

Figure S1. Effects of training on difficulty level of the perceptual task in the Control group (left panel) and Experimental group (right panel). Difficulty level refers to the average of the inverse of stimulus brightness in a given session. Error bars represent between-subjects SEM.

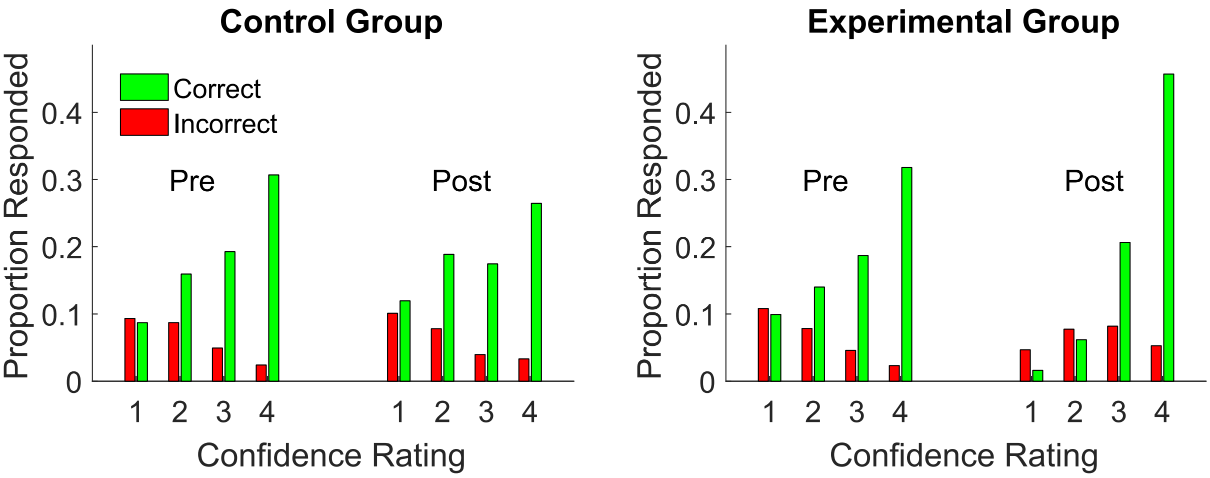

Figure S2. Change in confidence distributions due to training. Distributions of confidence ratings in the Control and Experimental groups at pre- and post-training for the perceptual task / trained stimulus type (see also Figure 4).

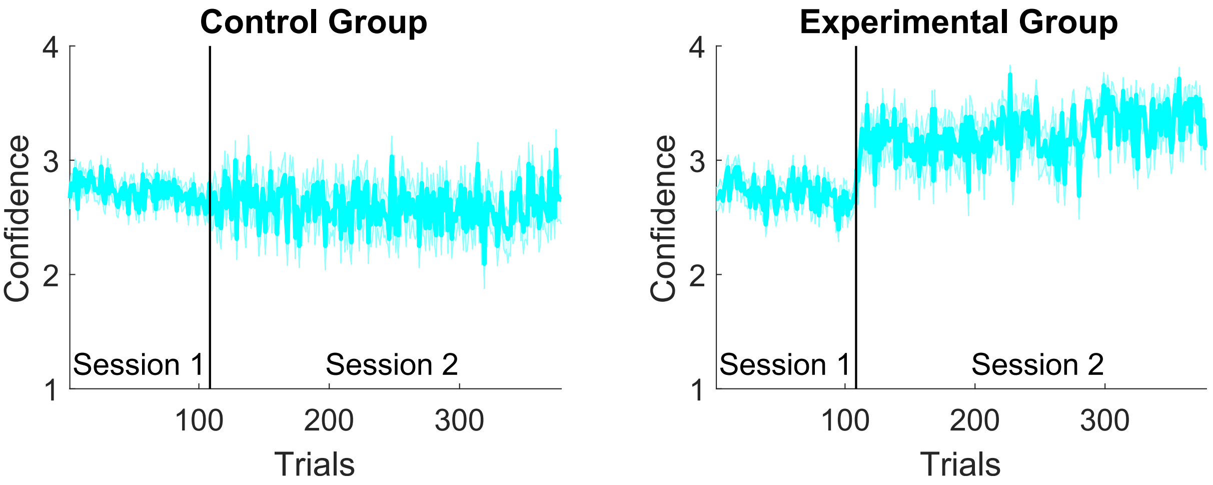

Figure S3. Timecourse of confidence change. Each panel shows the average trial-by-trial confidence rating across subjects for Sessions 1 and 2. The Experimental group shows an immediate shift upwards in confidence when exposed to the feedback schedule at the beginning of Session 2.

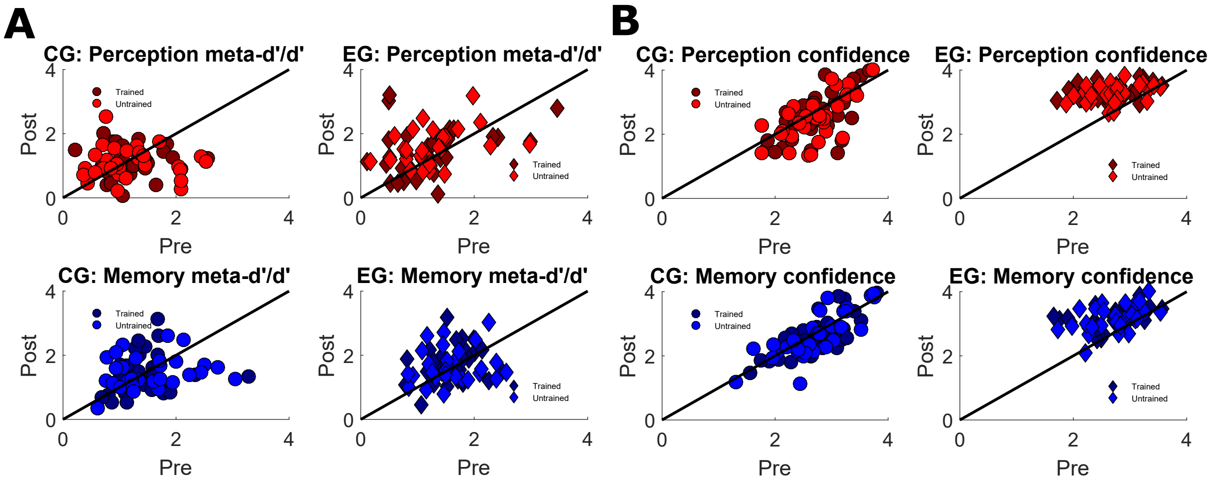

Figure S4. Scatter plots for the Control group (CG) and Experimental group (EG) showing changes from pre-training to post-training for metacognitive efficiency (A) and metacognitive bias / confidence level (B). Values above the solid black line indicate an increase, values below indicate a decrease. Points represent individual subjects. For ease of illustration two subjects with meta-*d'*/*d'* values > 4 are omitted from these plots.

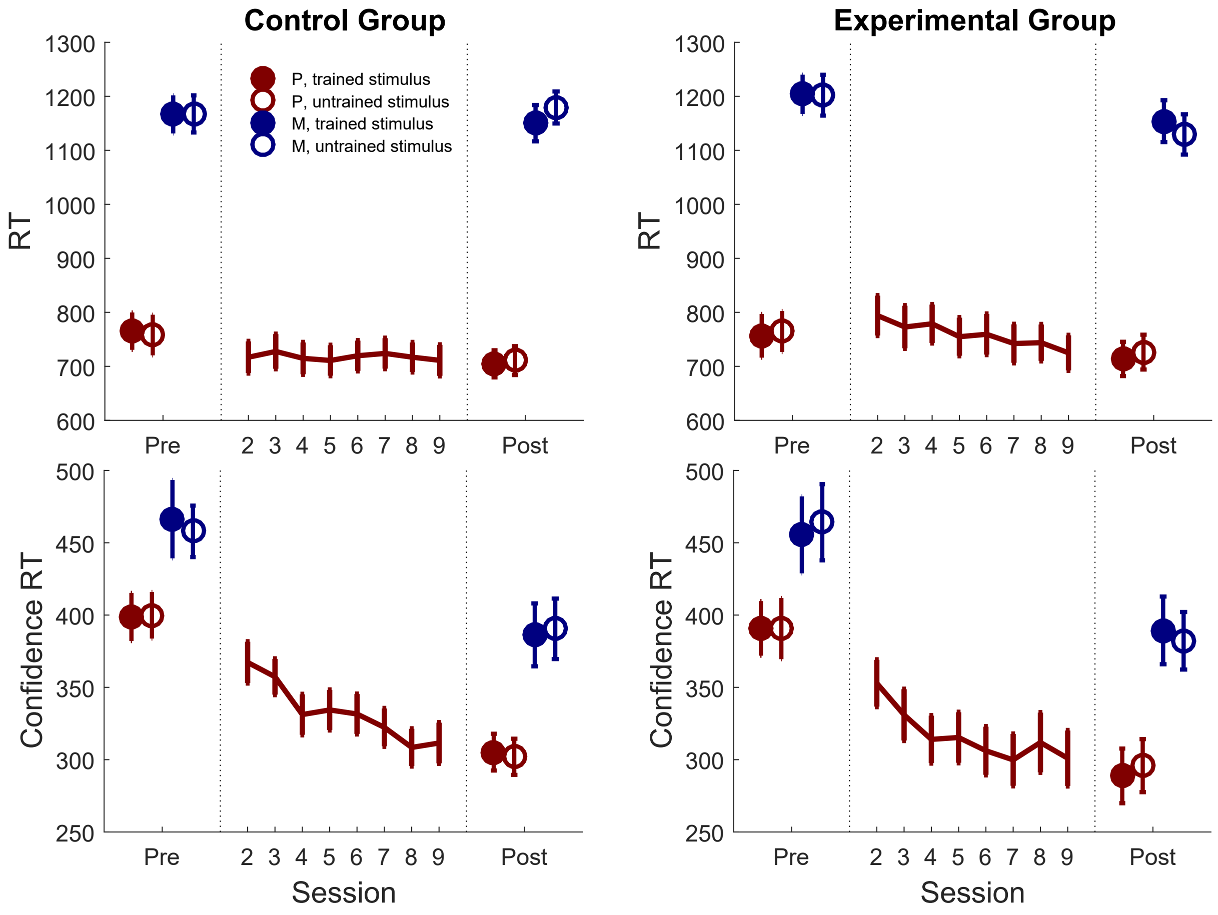

Figure S5. Mean response times for the type 1 decision (top row) and type 2 confidence report (bottom row) for the Control and Experimental groups. Error bars represent between-subjects SEM. P = perception; M = memory.

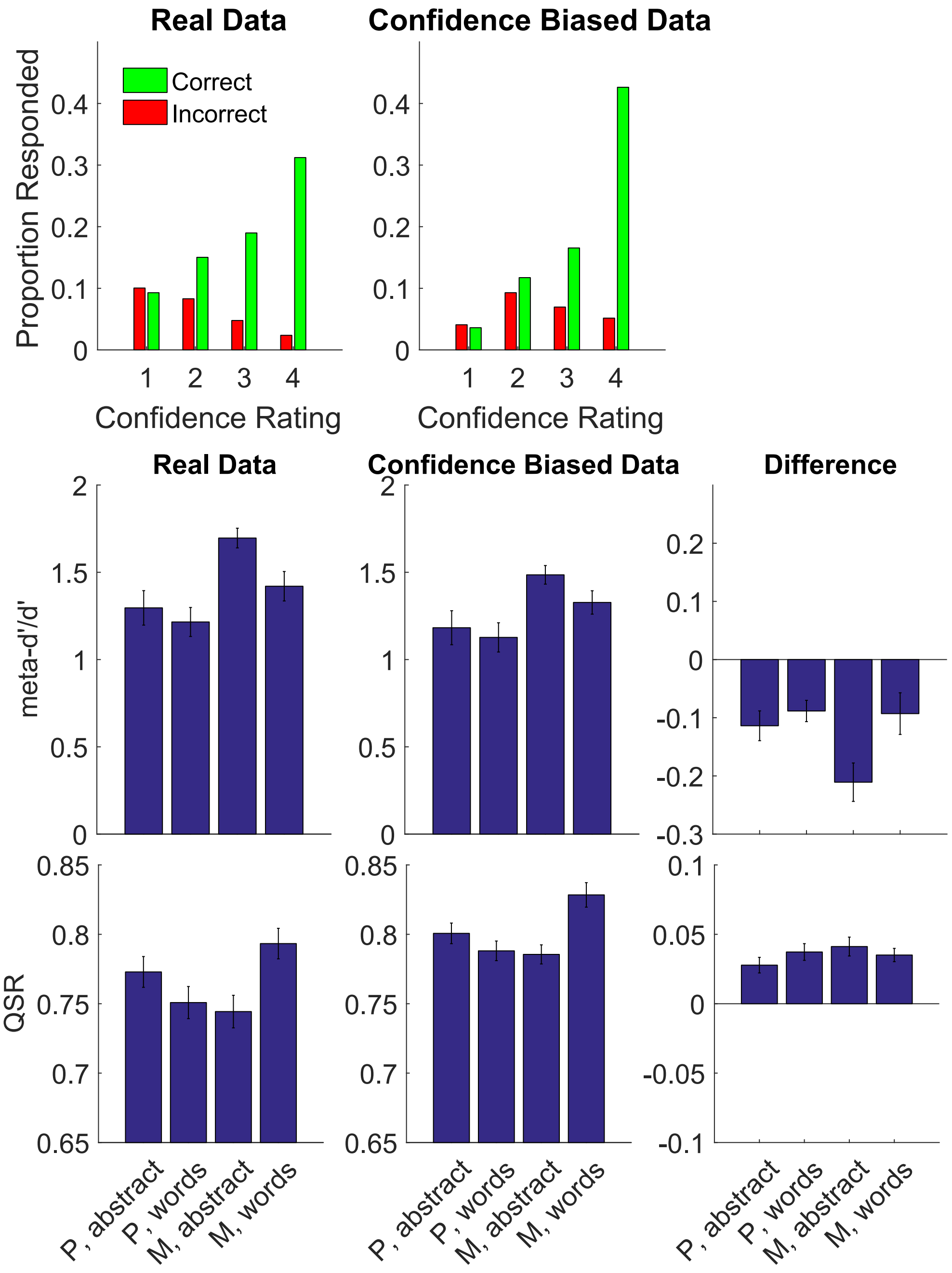

Figure S6. Comparison of pre-training data with simulated confidence-biased data. The confidence-biased data was created from the pre-training data set after randomly infusing a confidence increase of +1 with 0.6 probability into trials in which subjects did not already respond with maximum confidence. Artificial confidence bias leads to an increase in calibration score (QSR, lower row), as observed empirically, but a decrease in metacognitive efficiency (meta-d'/d', middle row) – the opposite of that observed empirically. Error bars represent between-subjects SEM.
